# Improved Stable Isotope Dilution Assay for Dietary Folates Using LC-MS/MS and Its Application to Strawberries

**DOI:** 10.3389/fchem.2018.00011

**Published:** 2018-02-06

**Authors:** Lisa Striegel, Soraya Chebib, Michael E. Netzel, Michael Rychlik

**Affiliations:** ^1^Chair of Analytical Food Chemistry, Technical University of Munich, Freising, Germany; ^2^Centre for Nutrition and Food Sciences, Queensland Alliance for Agriculture and Food Innovation, The University of Queensland, Brisbane, QLD, Australia

**Keywords:** stable isotope dilution assay, LC-MS/MS, folate, strawberry, processing, storage, retention

## Abstract

Folates play an important role in the human body and a deficiency of this vitamin can cause several diseases. Therefore, a reliable analytical method is crucial for the determination of folate vitamers in strawberries and other dietary folate sources. A stable isotope dilution LC-MS/MS method for analyzing folates in food was developed and validated. The folate vitamers Pteroylmonoglutamic acid, tetrahydrofolate, 5-methyltetrahydrofolate, and 5-formyltetrahydrofolate were quantified using ^13^C-labeled internal standards. Validation of the assay was accomplished by determining linearity, precision, recovery, limit of detection, and limit of quantification and revealed to be a precise, sensitive, and accurate method to determine folate vitamers. Strawberries are worldwide consumed and known to be a good dietary source of nutritive compounds. Using this method, folate concentrations in selected commercial strawberry cultivars and experimental breeding lines grown in Germany and Australia were investigated. Total folates varied from 59 to 153 μg/100 g on fresh weight basis. Furthermore, folate content after lyophilizing or freezing did not show any significant differences compared to fresh strawberries. However, significant losses of total folates in pureed strawberries could be observed after 5 days of storage with only 16% of the original concentration retained. In summary, some of the investigated strawberry cultivars/breeding lines can be considered as rich dietary sources of natural folates.

## Introduction

Folates, a generic term for a specific group of B vitamins play an essential role in different metabolic pathways. More specifically, tetrahydrofolate (H_4_folate) polyglutamates are acceptors and donors of one-carbons in a network of biosynthetic and catabolic reactions among primarily three different cellular compartments. The one-carbon metabolism in the cytoplasm catalyzes nucleotide biosynthesis as well as the remethylation of homocysteine to methionine. Secondly, folates are involved in the mitochondrial one-carbon metabolism generating amino acids and thirdly in the nucleus for DNA replication and repair (Schirch and Strong, 1989; Shane, 1989; Selhub, 2002). Therefore, these vitamers are essential for different metabolic pathways. However, the human body is not able to synthesize these compounds. If not sufficiently provided with foods or supplements, a lack of folate is mainly correlated with neural tube defects in newborns (van der Put and Blom, 2000) but can also result in decreased levels of methionine and increased levels of homocysteine. The latter is associated with cardiovascular diseases (Robinson, 2000) and Alzheimer‘s disease (Clarke et al., 1998; Snowdon et al., 2000; Terry et al., 2002). Moreover, there is already evidence for the association between folate status in relation to cancer risk, more specifically to colorectal cancer (Sanjoaquin et al., 2005). These indispensable functions and evidence of deficiencies in societies without mandatory fortification emphasize the importance of adequate folate supply and also underline the relevance of reliable analysis of folates in food.

A wide variety of vitamers, different numbers of glutamates attached with variations in the oxidation state in plants and not least the sensitivity when exposed to extreme pH values, heat, light and oxygen make the analysis of folates challenging. The most common used methods to quantitate folates are still microbiological assays. In addition, folates can also be quantified using protein-binding assays. However, for quantifying and distinguishing between the individual vitamers, liquid chromatography coupled with mass spectrometry is the method of choice. In this view, the application of stable isotope dilution assay (SIDA) has major advantages including the complete compensation for losses of analytes during extraction, for ion suppression during LC-MS/MS, and the so-called carrier effect (Rychlik and Asam, 2009). SIDA has proven its superiority during the last years and many research groups are using this state of the art methodology for folate analysis (Pawlosky et al., 2003; Pfeiffer et al., 2004; Ringling and Rychlik, 2013; de Ambrosis et al., 2016).

Folate intake mainly comes from fruits, vegetables, and cereal products.

Strawberries (*Fragaria x ananassa*) are tasty and worldwide popular and are an important dietary source of nutritive compounds such as folate and vitamin C, minerals, fiber, and especially polyphenolic phytochemicals (Giampieri et al., 2014; Afrin et al., 2016). The consumption of strawberries and derived products is reported to have many potential health benefits, for instance protection against inflammation, oxidative stress, type 2 diabetes, obesity, and cardiovascular disease (Giampieri et al., 2012, 2015; Afrin et al., 2016; Djurica et al., 2016). As already mentioned, strawberries can represent an important dietary source of natural folates, depending on cultivar, ripeness, and year of harvest, with values ranging between 30–69 μg/100 g fresh weight reported by Stralsjö et al. and 12.8–96 μg/100 g fresh weight reported by Tulipani et al. (Stralsjo et al., 2003; Tulipani et al., 2008). However, the folate content was determined by radioprotein-binding assay (Stralsjo et al., 2003) and microbiological assay (Tulipani et al., 2008) and was not determined by stable isotope dilution assay.

Reliable, accurate, and sensitive analytical methods are therefore crucial for the determination of individual folate vitamers in strawberries and other folate containing food sources. The aim of this work was the development and validation of a reliable and sensitive analytical method for the quantitation of the main folate vitamers in food, pteroylmonoglutamic acid (PteGlu), tetrahydrofolate (H_4_folate), 5-methyltetrahydrofolate (5-CH_3_-H_4_folate), and 5-formyltetrahydrofolate (5-CHO-H_4_folate) using strawberries as the test-matrix and additionally analyzing strawberries grown under different conditions. Furthermore, the loss of folates during strawberry processing and storage was also studied in pilot trials.

## Materials and methods

### Samples and study design

A diverse set of commercial strawberry cultivars and experimental breeding lines was investigated in the present study: the cultivars inter alia Malvina, Florenz, Salza, Royal, Santana, and Elsanta were obtained from three different commercial growers in Germany (Freising), white strawberry breeding lines (P100120; P100677; P100-696; F.nilgerrensis Mt. Leigong 2) from the Chair of Biotechnology of Natural Products, Centre of Life Sciences Weihenstephan, Technical University Munich (TUM), Germany as well as one commercial strawberry (Festival) from Australia (Queensland Government, Department of Agriculture and Fisheries, Research Station Nambour, QLD, Australia).

All cultivars and breeding lines were harvested at the eating ripe stage. Once harvested they were transported to the Chair of Analytical Food Chemistry, TUM respectively to the Centre for Nutrition and Food Sciences, University of Queensland, Coopers Plains, for sample preparation and analysis. The German strawberry samples were thoroughly blended with a commercial hand mixer and extracted. As the Australian grown strawberries had to be shipped to Germany for analysis, folate stability during transportation was assessed. Freeze-drying and short storage at room temperature proved to have no significant effect on folate content (see below), thus the Australian grown strawberries were freeze-dried and milled using a Retsch MM301 cryogenic mill (Retsch, Haan, Germany) before they were sent to TUM for extraction and analysis. The white strawberries were also received freeze dried from the Chair of Biotechnology of Natural Products. The moisture content was determined for the Australian grown and the white strawberries. The strawberries were weight before and after freeze drying and the moisture content was calculated in percent.

Commercial strawberries from a local supermarket in Munich were used to assess the effect of processing and storage on folates. Processing: after thorough mixing, one randomly selected sample was immediately extracted. Sample aliquots were frozen and freeze-dried, respectively, and stored in the dark at −20°C until analysis. Storage: samples of the blended strawberries were randomly selected and stored in the dark at 7°C until analysis whereas a sample aliquot was immediately extracted.

### Chemicals

Acetonitrile, methanol, and water (analytical grade) were purchased from VWR (Ismaning, Germany); ascorbic acid, formic acid (>95%), and 2-(N-morpholino)-ethanesulfonic acid (MES) from Sigma-Aldrich (Steinheim, Germany); potassium dihydrogen phosphate, sodium acetate trihydrate, and sodium hydroxide from Merck (Darmstadt, Germany); disodium hydrogen phosphate (anhydrous) and sodium chloride from Alfa Aesar and Baker J.T. (Thermo Fisher, Karlsruhe, Germany); rat serum and chicken pancreas containing γ-glutamyl hydrolase (EC 3.4.19.9) from Biozol (Eching, Germany) and Difco (Sparks, MD, USA), respectively and dithiothreitol (DTT) was purchased from Applichem (Darmstadt, Germany).

Both the unlabeled reference compounds (H_4_folate, 5-CH_3_-H_4_folate, 5-CHO-H_4_folate, and 10-CHO-PteGlu) and isotope labeled internal standards ([^13^C_5_]-PteGlu, [^13^C_5_]-H_4_folate, [^13^C_5_]-5-CH_3_-H_4_folate, and [^13^C_5_]-5-CHO-H_4_folate) were acquired from Schircks Laboratories (Jona, Switzerland), whereas PteGlu was obtained from Fluka (Sigma-Aldrich, Steinheim, Germany). Strata strong anion exchange (SAX) cartridges (quaternary amine, 500 mg, 3 mL) were purchased from Phenomenex (Aschaffenburg, Germany).

### Solutions

#### Buffer solution for extraction

Buffer containing 2 g/L ascorbic acid and MES (200 mmol/L) solution with 0.1 g/L DTT was adjusted to pH 5 with 7.5 M NaOH. Phosphate buffer (100 mmol/L) was prepared by setting a 100 mmol/L disodium hydrogen phosphate aqueous solution with an potassium dihydrogen phosphate (100 mmol/L) aqueous solution to pH 7.0.

#### Equilibration buffer for SPE clean-up

Buffer consisting of 0.2 g/L DTT and 10 mmol/L phosphate buffer was used for the SAX cartridges.

#### Elution buffer for SPE clean-up

Buffer was prepared by mixing 5% aqueous sodium chloride, 100 mmol/L aqueous sodium acetate, 0.1 g/L DTT and 1 g/L ascorbic acid.

#### Enzyme solutions for deconjugation

The chicken pancreas was prepared by adding 30 mg lyophilized chicken pancreas to 30 mL aqueous phosphate buffer solution (100 mmol/L) and 1 g/L ascorbic acid adjusted to pH 7. Rat serum was used without further dilution.

#### Stock solutions of analytes and internal standards

The stock solutions of the reference material were prepared freshly before each sample extraction by first dissolving 10 mg of PteGlu in 100 mL of MES, presolved in 10 mL of phosphate buffer and 2 mg of H_4_folate, 5-CH_3_-H_4_folate, 5-CHO-H_4_folate, and 10-CHO-PteGlu in 10 mL MES, and presolved in 3 mL of phosphate buffer. The purity of the unlabeled analytes was determined before each extraction by HPLC/DAD using PteGlu as internal standard (ISTD). The stock solutions were diluted 1:20 for the LC-MS/MS response. With the known concentration of the unlabeled reference material the concentration of the isotopical internal standards can be determined. The isotope labeled internal standards [^13^C_5_]-PteGlu, [^13^C_5_]-H_4_folate, [^13^C_5_]-5-CH_3_-H_4_folate, and [^13^C_5_]-5-CHO-H_4_folate were once dissolved in concentrations of 60–70 μg/mL in extraction buffer. For the sample extraction the ISTDs were further diluted to final concentrations of 8–11 μg/mL suitable for addition during extraction. All internal standard solutions were stored at −20°C in the dark. The stability of the internal standard stock solutions has to be verified during each extraction. For that, a mixture of unlabeled and labeled reference material was prepared freshly and the concentration of internal standards was determined new during each extraction.

### Sample preparation

The sample extraction was performed under subdued light. Initially homogenized food samples (50 mg of strawberries on dried weight basis, 300 mg of strawberries on fresh weight basis) were weighed into Pyrex bottles and were equilibrated for 15 min with 10 mL buffer. Internal standards were added to samples in amounts adjusted to the expected contents of the respective analytes to fall in the given calibration range. Further, the samples were stirred for 15 min for equilibrating and boiled for 10 min. Once the samples were cooled on ice, 2 mL chicken pancreas suspension and 0.8 mL rat serum were added for deconjugation. After overnight incubation with a minimum of 12 h in a water bath at 37°C, the samples were heated in boiling water for 10 min, cooled on ice, transferred into centrifuge tubes with 10 mL acetonitrile and centrifuged for 20 min (4000 rpm, 4°C). The supernatant with the aqueous and organic phase (approximate 20 mL) was then purified by strong anion-exchange (SAX, quaternary amine, 500 mg, 3 mL) solid-phase extraction (SPE). The cartridges were activated with two volumes of methanol and equilibrated with two volumes of buffer. After applying the extracts the cartridges were washed again with three volumes of equlibration buffer and run dry. The folates were eluted using 2 mL elution buffer. The final eluate was membrane filtered (PVDF, 0.22 μm) and measured by LC-MS/MS. Additionally, for each batch of rat serum and chicken pancreas, the endogenous amount of folates from the enzymes added was determined as described above and substracted from the measured total folate of the samples.

### Instrumental conditions

#### HPLC-DAD

The quantitation of the solutions of unlabeled analytes was performed on a Shimadzu HPLC/DAD system (Shimadzu, Kyoto, Japan) using a reversed phase column (C18 EC, 250 × 3 mm, 5 μm, 100 Å, precolumn: C18, 8 × 3 mm, Machery-Nagel, Düren, Germany) for separation. The mobile phase consisted of (A) 0.1% acetic acid and (B) methanol delivered as a binary gradient at a flow rate of 0.4 mL/min. Gradient elution started at 10% B for 7 min, followed by raising the concentration of B lineary to 50% during the next 14 min. Subsequently, the gradient was linearly programmed to 100% B within 2 min, and then maintained at 100% B for 1 min. Next, the mobile phase returned to 10% B within 2 min and was equilibrated for 9 min before the next run. The injection volume was 10 μL and the analysis was done at room temperature.

#### LC-MS/MS

LC-MS/MS was carried out on a Shimadzu Nexera X2 UHPLC system (Shimadzu, Kyoto, Japan) with a Raptor ARC-18 column (2.7 μm, 100 × 2.1 mm, Restek, Bad Homburg, Germany) and a Raptor ARC-18 precolumn (2.7 μm, 5 × 2.1 mm, Restek, Bad Homburg, Germany) as a stationary phase that was kept at 30°C. The mobile phase consisted of (A) 0.1% formic acid and (B) acetonitrile with 0.1% formic acid delivered as a binary gradient at a flow rate of 0.4 mL/min. The gradient started at 3% B, then was raised linearly from 3 to 10% B during the next 2.5 min, and then maintained at 10% B for 2.5 min. Next, the mobile phase was increased to 15% B within 5 min and to 50% within a further 1 min. Subsequently, the mobile phase was held at 50% for 1 min before equilibrating the column for 4 min with the initial concentration of 3% B. The injection volume was 10 μL.

The LC was interfaced with a triple quadrupole ion trap mass spectrometer (LCMS-8050, Shimadzu, Kyoto, Japan). It operated in the positive ESI mode for all analytes. The ion source parameters were set as follows: heat block, dilution line, and interface temperature were set to 400, 250, and 300°C, respectively, drying gas, heating gas, and nebulizing gas flow were set to 10, 10, and 3 L/min, respectively, collision-induced dissociation gas was applied to 270 kPa, and interface voltage was applied to 4 kV. MS parameter were optimized by injection of each unlabeled standard solution (1 μg/mL). The optimized MS parameters were assumed for the respective isotope-labeled internal standards. The mass spectrometer was operated in the multiple reaction monitoring (MRM) mode for MS/MS measurements at the conditions detailed in Table 1. The first MRM transition was the target ion used for quantitation, whereas the second MRM transition represents the qualifier ion. The relative abundance of the qualifier ions to target ions of the analytes in the matrix compared to pure standard solutions verifies the analytes and peak purity. A waste valve diverted the column effluent to the mass spectrometer just from 2.1 to 7 min. Data acquisition was performed with LabSolutions software 5.8 (Shimadzu, Kyoto, Japan).

**Table 1:** MRM scan parameters for PteGlu, H_4_folate, 5-CH_3_-H_4_folate, 5-CHO-H_4_folate, and the respective internal standards.

**Compound**	**Precursor [m/z]**	**Product [m/z]**	**Dwell time [ms]**	**Q1 Pre bias [V]**	**CE [V]**	**Q3 Pre bias [V]**
PteGlu	442.30	295.15	50.0	−13.0	−16.0	−16.0
		176.20	50.0	−13.0	−37.0	−20.0
		120.05	50.0	−13.0	−35.0	−14.0
[^13^C]-PteGlu	447.2	295.15	50.0	−13.0	−16.0	−16.0
		176.20	50.0	−13.0	−37.0	−20.0
		120.05	50.0	−13.0	−35.0	−14.0
H_4_Folat	446.0	299.20	50.0	−22.0	−20.0	−16.0
		120.10	50.0	−22.0	−37.0	−14.0
		166.15	50.0	−22.0	−41.0	−19.0
[^13^C]-H_4_Folat	451.3	299.20	50.0	−22.0	−20.0	−16.0
		120.10	50.0	−22.0	−37.0	−14.0
		166.15	50.0	−22.0	−41.0	−19.0
5-CH_3_-H_4_Folat	460.2	313.20	50.0	−13.0	−20.0	−17.0
		180.15	50.0	−13.0	−37.0	−14.0
		194.25	50.0	−23.0	−33.0	−22.0
[^13^C]-5-CH_3_-H_4_Folat	465.3	313.2	50.0	−13.0	−20.0	−17.0
		180.15	50.0	−13.0	−37.0	−14.0
		194.25	50.0	−23.0	−33.0	−22.0
5-CHO-H_4_Folat	474.3	327.15	50.0	−14.0	−20.0	−17.0
		299.20	50.0	−14.0	−31.0	−16.0
		208.20	50.0	−18.0	−36.0	−24.0
[^13^C]-5-CHO-H_4_Folat	479.25	327.15	50.0	−14.0	−20.0	−17.0
		299.20	50.0	−14.0	−31.0	−16.0
		208.20	50.0	−18.0	−36.0	−24.0

### Method validation

#### Calibration and quantitation

For the response curves constant amounts of internal standard (S) were mixed with varying amounts of analyte (A) in molar ratios [n(A)/n(S)] between 0.03 and 14.9 preparing 12–13 calibration points. Linear and polynomial regression was used by combining the molar ratios [n(A)/n(S)] with the peak areas [A(A)/A(S)] from the LC-MS/MS measurements.

#### Limits of detection and quantification

Limits of Detection and Quantification (LOD, LOQ) were determined according to Vogelgesang and Hadrich (1998). A mixture of pectin and sugar in similar proportions as present in strawberries and treated under UV light and repeatedly frozen and thawed was used as a blank matrix. The blank matrix was analyzed to confirm the absence of folates. The matrix was spiked with the unlabeled analytes at four different amounts with the lowest concentration slightly above the estimated LOD (three times higher than the backround signals) and the highest concentration 10-fold higher. Each concentration level was determined in triplicate. Then the data obtained from the SIDAs and spiked amounts were correlated. A subsequent regression calculation provided the calibration line and the confidence interval, which was used to compute the LODs and LOQs according to the published method (Vogelgesang and Hadrich, 1998).

#### Precision

Natural strawberries were used for precision measurements. For determining the inter-day precisions strawberry samples were analyzed in triplicate, with three independent experiments over 2 weeks. Inter-injection assay was determined by injecting one sample three times in a row. For determining the intra-day precision the same strawberry sample was extracted in triplicate.

#### Recovery of SIDAs

Blank matrices were spiked in triplicate with all vitamers in three different amounts of analytes (1.60, 9.80, 97.7 μg/100 g for PteGlu; 10.1, 26.8, 90.4 μg/100 g for H_4_folate; 1.60, 42.7, 108 μg/100 g for 5-CH_3_-H_4_folate, and 5.10, 15.6, 41 μg/100 g for 5-CHO-H_4_folate). The samples were analyzed as already described and the recoveries were calculated as the ratio of the detected and spiked contents.

## Results and discussion

### Essential adjustments of the current stable isotope dilution assay

In course of continuous improvements of the initial SIDA by including additional folate vitamers and simultaneous monitoring of deconjugation, the following modifications of the analytical procedure were necessary (Ringling and Rychlik, 2017a). Firstly, the need for cutting down the long run times of 45 min required performing LC on a UHPLC-system and secondly the switch to the commercially available ^13^C-labeled folates used by many other groups (Pawlosky et al., 2003; Chandra-Hioe et al., 2011). Of these, ^13^C-labeled PteGlu, H_4_folate, 5-CH_3_-H_4_folate, and 5-CHO-H_4_folate are commercially available, but ^13^C-labeled 10-CHO-PteGlu is not. Therefore, we set up a response function using ^13^C-5-CHO-H_4_folate as internal standard for 10-CHO-PteGlu and calculated the content of 10-CHO-PteGlu in strawberries. We found the content of 10-CHO-PteGlu below its LOD and decided the validation of 10-CHO-PteGlu not to be crucial for the determination of total folate content in strawberries. The vitamers PteGlu, H_4_folate, 5-CH_3_-H_4_folate, and 5-CHO-H_4_folate were validated and taken in account when calculating total folates in the strawberry samples under study.

### Developing a time efficient LC-MS method

**Figure 1:**
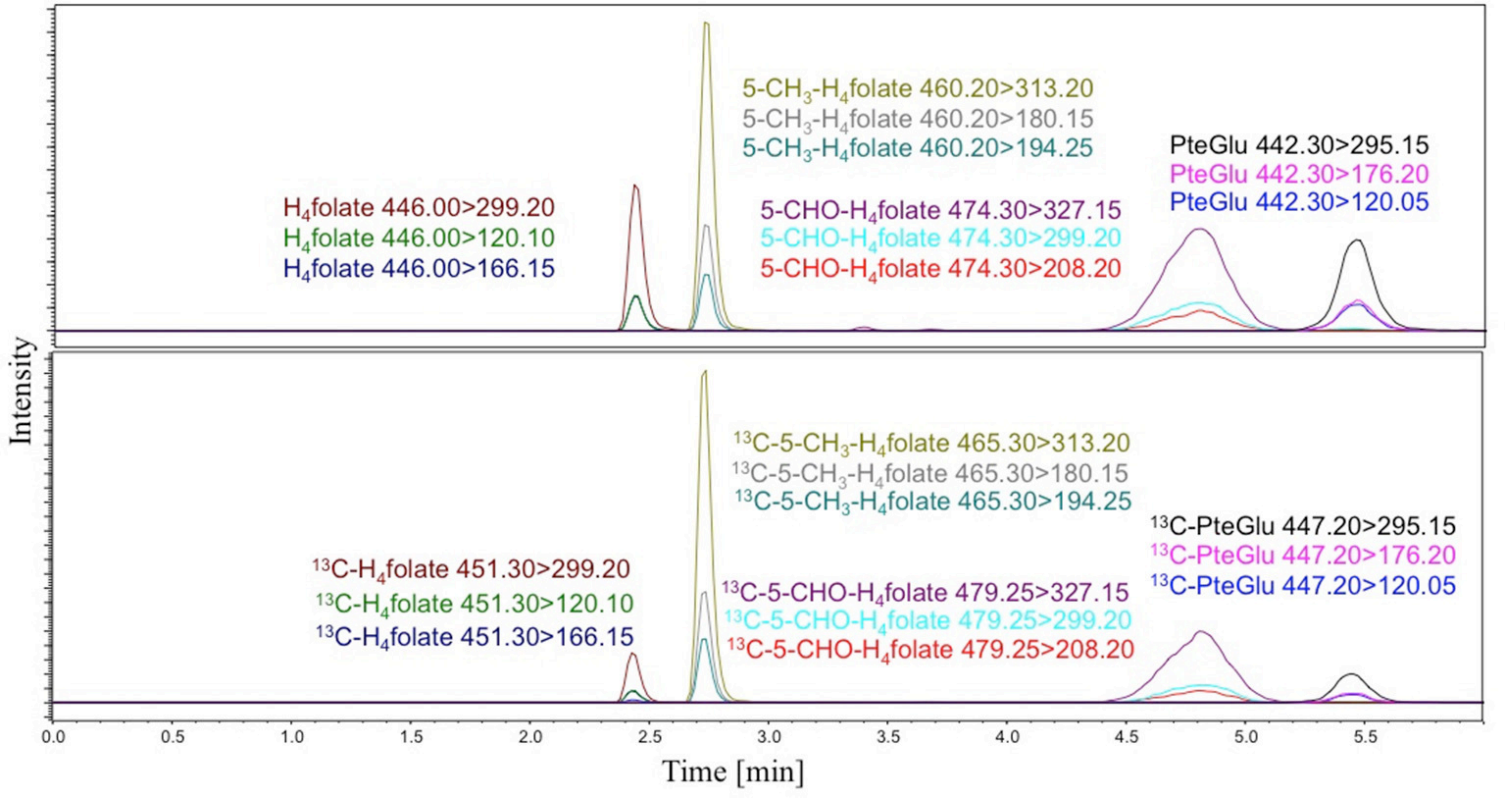
LC-MS/MS-chromatograms with H_4_folate, 5-CH_3_-H_4_folate, and PteGlu and the respective internal standards.

The developed system enabled an elution and baseline separation of all analytes within 6 min and thus enabled a high throughput of samples. The development of the LC system is described in Supplementary materials. A representative LC-MS/MS-chromatogram of all analytes and the respective internal standards is shown in Figure 1. The late-eluting peaks 5-CHO-H_4_folate and PteGlu show broad peaks. The broad and unresolved peak of 5-CHO-H_4_folate is due to interconversion on the column in acidic conditions to 5,10-CH^+^-H_4_folate (Robinson, 1971). PteGlu shows also a broad peak shape as we need a flat gradient to separate it completely from the other folates and interferences.

The sample extraction was carried out as described by Ringling and Rychlik (2013) with slight modifications. As the internal standards appear to be degraded after being added directly to the freeze-dried samples, two equilibration steps were found to be crucial: an initial 15 min equilibration of the samples with MES-buffer before adding the internal standard followed by a second equilibration of another 15 min (more details are described in Supplementary materials).

According to Ringling and Rychlik (2017a), a double enzyme treatment consisting of chicken pancreas and rat serum was used for extraction and deconjugation. They investigated the necessity of a double enzyme and triple enzyme treatment, however, a further treatment step with amylase or protease showed no benefits compared to a simple double enzyme treatment (Ringling and Rychlik, 2017a). For our experiments, to achieve a complete deconjugation the sample weight and the amount of enzymes had to be adjusted. A complete deconjugation was achieved by using 2 mL of chicken pancreas (3 mg/3 mL) and 0.8 mL rat serum. Furthermore, to fall in the given calibration line the sample size of fresh strawberries was adjusted to 300 mg and that of freeze dried strawberries to 50 mg. To monitor the deconjugation process folate diglutamates and higher folate polyglutamates were included in the LC-MS/MS method (Ringling and Rychlik, 2017a).

### Validation

#### Calibration of SIDAs

Response functions for all vitamers were obtained using linear and polynomial regression. The linearity test of Mandel confirmed the linearity of PteGlu and H_4_folate and resulted in a polynomial regression line for 5-CH_3_-H_4_folate and 5-CHO-H_4_folate, respectively. Calibration curves of PteGlu and H_4_folate were linear within the molar ratios $\frac{n\left(A\right)}{n\left(S\right)}$ between 0.1 and 14.9 for PteGlu (*y* = 0.9211*x* + 0.0375, *R*^2^ = 0.9993) and between 0.03 and 9.11 for H_4_folate (*y* = 0.247*x* + 0.0218, *R*^2^ = 0.9997). However, calibration curves of 5-CH_3_-H_4_folate and 5-CHO-H_4_folate were polynomial within the molar ratios $\frac{n\left(A\right)}{n\left(S\right)}$ between 0.03 and 10.1 for 5-CH_3_-H_4_folate (*y* = 0.0034*x*^2^ + 0.7991*x* − 0.0015, *R*^2^ = 0.9999) and between 0.05 and 9.60 for 5-CHO-H_4_folate (*y* = 0.0251*x*^2^ + 1.107*x* + 0.0538, *R*^2^ = 0.9975). The calculations of the response equations are explained in the Supplementary materials.

#### LODs and LOQs

The LODs and LOQs were determined according to Vogelgesang and Hadrich (1998) and the results are shown in Table 2. The LODs range between 0.17 and 0.33 μg/100 g and the LOQs range between 0.51 and 0.96 μg/100 g. The values are slightly higher than that of our previous SIDA using either bread as a folate free matrix or labeled standards for spiking (Ringling and Rychlik, 2013), but still satisfactory to detect even low folate contents in foods. Folates are always present in real food matrices, therefore a self composed matrix similar to strawberries was chosen for validation. As a folate free matrix, a mixture of pectin and sugar was used that had been depleted from folates by treatment with UV-light and repetitive freezing and thawing. However, all analytes could still be detected in very small amounts and were subtracted from the spiked matrices. To avoid a residual folate content in the matrix Ringling and Rychlik (2013) used either pure buffer solution as a folate-free matrix or spiked the matrix with labeled standards instead of unlabeled analytes, which could result in lower values. Furthermore, the LODs and LOQs are calculated in relation to the sample weight. Ringling and Rychlik used 0.5–2 g of sample weight, whereas we were able to reduce the sample weight to 0.05–0.3 g. Therefore, the method enables a higher sensitivity (Ringling and Rychlik, 2013).

**Table 2:** Validation data for the stable isotope dilution assay.

**Analyte**	**LOD**	**LOQ**	**Precision (*****n*** = **3) [RSD%]**			**Recovery [%]**		
	**[μg/100 g]**	**[μg/100 g]**	**Inter-injection**	**Intra-day**	**Inter-day**	**Spiking level 1 (low)**	**Spiking level 2 (medium)**	**Spiking level 3 (high)**
PteGlu	0.33	0.96	3.82^a^	2.70	4.83^a^	109	114	105
H_4_folate	0.25	0.76	4.46	2.44	5.06	99.1	99.4	99.3
5-CH_3_-H_4_folate	0.17	0.51	1.92	2.74	3.04	96.7	99.5	101
5-CHO-H_4_folate	0.32	0.93	2.49	4.60	4.83	81.9	98.2	96.7

*The LODs and LOQs were determined in a matrix of pectin and sugar and were related to a sample weight of 50 mg. The precision values were determined with strawberries and matrix of pectin and sugar showing different folate compositions*.

a *For determining the inter-day and intra-day precisions the matrix of pectin and sugar was used*.

#### Precision

The inter-injection and inter- and intra-day precisions are shown in Table 2. The final inter-injection assay varied between 1.92 and 4.46%. After adjusting the protrusion of the ESI needle we were able to stabilize the ionization of the analytes and improve the inter-injection assay from 3.74, 5.12, 4.88 to 4.82% for PteGlu, H_4_folate, 5-CH_3_-H_4_folate, and 5-CHO-H_4_folate to the final results shown in Table 2. The intra-day precision of all analytes varied between 2.44 and 4.60%. The inter-day precision ranged between 3.04 and 5.06%. The best precisions showed 5-CH_3_-H_4_folate, which is obviously due to the highest amounts of this vitamer in strawberries. The precisions presented here were similar or even better than those previously reported for SIDAs (Vishnumohan et al., 2011; Ringling and Rychlik, 2013).

#### Recoveries of SIDA

The recoveries of all analytes for three different spiking levels varied between 92.3 and 110% and were in the same range as previously reported for other food matrices (Table 2) (Vishnumohan et al., 2011; Ringling and Rychlik, 2013).

### Analysis of folates in strawberries

**Figure 2:**
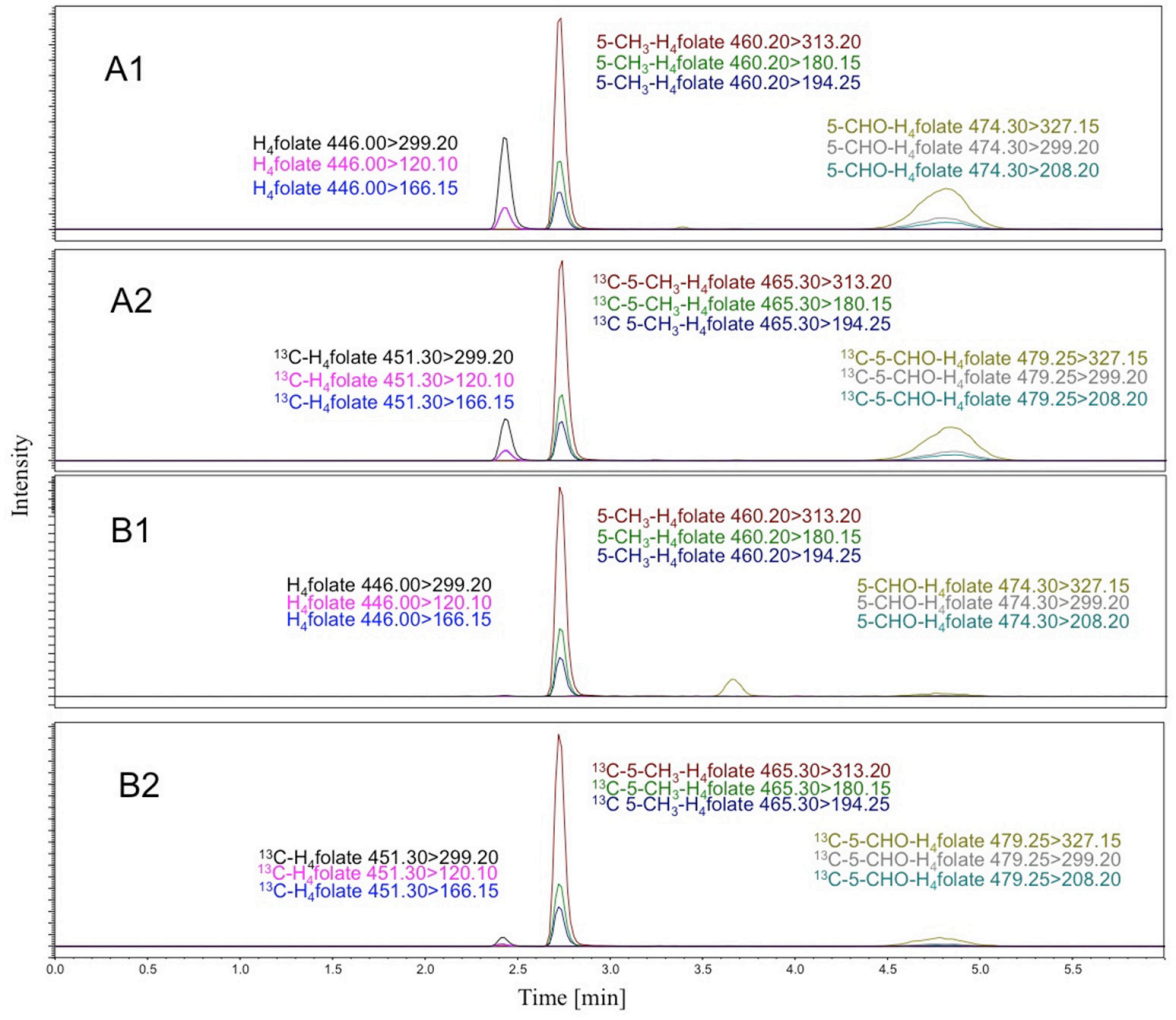
LC-MS/MS-chromatogram of a strawberry sample (**B1**: MRMs for analytes; **B2**: MRMs for internal Standards) and the response run of pure analyte-internal standard mixtures (**A1**: MRMs for analytes; **A2**: MRMs for internal Standards).

All folate results are means of triplicates ± standard deviation. The water content of strawberries (Festival) of 92% before freeze drying was taken into account and the folate concentrations were calculated on fresh weight basis. A representative LC-MS/MS-chromatogram of a strawberry sample and the appropriate response is shown in Figure 2.

#### Commercial strawberry cultivars and experimental breeding lines

**Figure 3:**
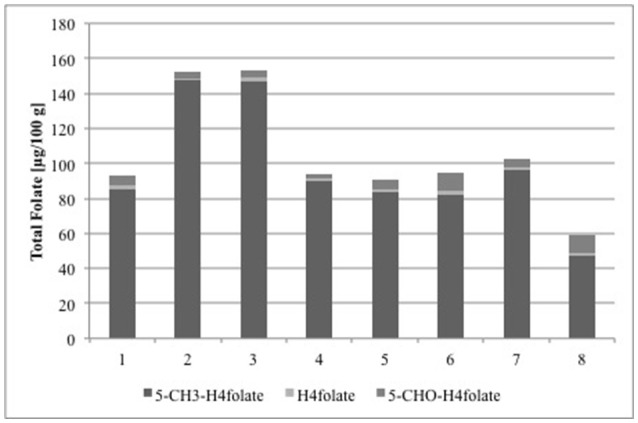
Vitamers and total folate content calculated as PteGlu in [μg/100 g] of three strawberry cultivars grown in Germany (1: cultivar 1, 2: cultivar 2, and 3: cultivar 3), an Australian cultivar grown in Queensland (4: Festival), and white strawberry breeding lines grown in Germany (5: P100120, 6: P100677, 7: P100696, and 8: F. nilgerrensis Mt. Leigong 2).

To prove the applicability of the new method, folates were analyzed in strawberries grown upon different environmental conditions. As the samples had to be transported up to more than 24 h between sampling and analysis, the most suitable procedure for preserving the folate content had to be elaborated. Freeze-drying proved to have no significant impact (*p* > 0.05) on folate amount (Table 3) and even storing the freeze-dried samples for 24 h did not alter the folate content. Therefore, the analysis of strawberries from the different locations can be compared directly with each other and the results are shown in Figure 3. The German grown strawberry cultivars, cultivar 2 and cultivar 3 (Figure 3), had almost the same total folate content (152 ± 4.0 and 153 ± 5.0 μg/100 g), whereas folates in cultivar 1 were considerable lower (93.0 ± 5.3 μg/100 g). However, the three cultivars were grown in the same area 50 km north of Munich under the same environmental conditions. Total folate content in Festival grown under subtropical conditions 100 km north of Brisbane (Australia) was 93.7 ± 6.5 μg/100 g. The main vitamer in all samples was 5-CH_3_-H_4_folate, whereas H_4_folate and 5-CHO-H_4_folate appeared only in small amounts. PteGlu was below the LOQ (0.96 μg/100 g) and therefore not taken into account when calculating the total folate content. Different folate levels in various strawberry cultivars and breeding lines have already been reported previously (Stralsjo et al., 2003; Tulipani et al., 2008). Stralsjö et al. (2003) found folate contents from 30 to 69 μg/100 g, whereas Tulipani et al. (2008) reported folate concentrations of up to 100 μg/100 g. Bioactive components such as phytochemicals and vitamins in naturally grown fruits are dependent on many factors as climate, soil and also cultivar, ripeness, and year of harvest (Hägg et al., 1995). However, the found differences in the total folate content of German grown strawberries cannot be explained by the climate. The Australian grown strawberries and the German cultivar 1, even though they grew under different climate conditions, showed similar total folate. Based on these results, we cannot hypothesize a relationship between the folate content and the origin of strawberries. Furthermore, Reganold et al. found that organic and conventional growing can have a significant impact on the composition of strawberries and that organic strawberries were of higher quality when considering the vitamin C and total phenolic content (Reganold et al., 2010). In this respect, variations of vitamin C in strawberries can affect the folate content as a higher vitamin C content can lead to an increased stability of folates (Wilson and Horne, 1983). This assumption was also confirmed by Ringling and Rychlik, who performed *in vivo* studies to simulate food folate digestion and found out that ascorbic acid stabilizes folates, particularly 5-CH_3_-H_4_folate during digestion. The addition of ascorbic acid in physiological amounts improved the stability of some folates depending on the food matrix (Ringling and Rychlik, 2017b).

**Table 3:** Total folate content calculated as PteGlu in [μg/100 g] of fresh strawberries compared to frozen and lyophilized strawberries and the relative folate retention.

	**Untreated**	**Frozen**	**Lyophilized**
Total folate [μg/100 g]	115 ± 2.0	107 ± 7.9	97.7 ± 6.4
Relative folate retention [%]		92.8 ± 7.4	91.3 ± 6.8

We also analyzed a selection of white strawberry breeding lines and determined folate levels of 59.2 ± 0.9, 90.6 ± 1.1, 95.0 ± 0.3, and 102 ± 0.9 μg/100 g (Figure 3), which is in the same range as for the red colored cultivars. Furthermore, the vitamer distribution was similar to that in the red strawberries with 5-CH_3_-H_4_folate as the main vitamer and H_4_folate and 5-CHO-H_4_folate as the minor ones. As can be anticipated from their different colors, a different phenolic composition of commercial red and white strawberries were reported in recent studies on fruits grown in Chile (Simirgiotis et al., 2009). As far as we know we are here the first to report folate analysis of white strawberries, which allows the assumption that folates are not linked to the phenolic composition of strawberries. Moreover, to the best of our knowledge, this is the first report about folate concentrations in this specific selection of white and red colored strawberry cultivars and breeding lines grown in Germany and Australia.

#### Effect of storage

**Figure 4:**
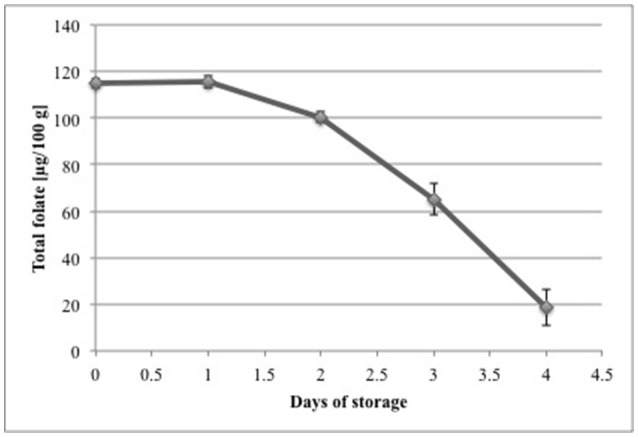
Folate retention during storage of fresh, blended strawberries of a commercial cultivar at 7°C in the dark. Total folate concentrations are calculated as PteGlu in [μg/100 g].

The retention of folates in strawberry puree stored in the dark at 7°C is shown in Figure 4. After the second day no losses of folates were observed. The retention of folates after 3 days was 87 and 57% after 4 days. After 5 days of storage only 16% of the initial folate concentration on day 1 was left. Starting from the third day we calculated a significant (*p* < 0.05) difference compared to day 1. We observed a higher relative loss of folates compared to Stralsjö et al. (2003). They found retention of 84% after 3 days and still 70% after 9 days, whereas in our study only 57% of total folate was left after 4 days. However, Stralsjö et al. (2003) stored the whole strawberries and took subsamples, whereas we stored the blended (pureed) samples to achieve better homogeneity of the fruit matrix and to ensure representativeness of sampling. The stability of blended strawberries compared to intact fruits can vary significantly. Folates can be encapsulated in plant cells or subcellular components and blending disrupts cell wall structures, which can result in an expedited loss of folates due to their susceptibility to react with other matrix components such as enzymes. The results gave a first impression of the stability of strawberries during storage and showed that there is a need to perform further storage tests. Therefore, future storage trials should include whole strawberry fruit to better mimic commercial handling and conditions.

## Conclusion

The SIDAs presented here are reliable and sensitive methods for analyzing individual folate vitamers in strawberries and other foods showing similarly good validation data as those from previously reported SIDAs. Due to the reduction of the sample weight, the method shows a higher sensitivity. Furthermore, the implementation of UHPLC enables high-throughput of samples. The method enables the accurate analysis of the total folate content and the vitamer distribution in different cultivars as described above as well as the progress monitoring of strawberries during storage or processing. In further studies, the method can be used to investigate strawberries regarding to climate changes, ripening stages, year of harvest, and to screen specific strawberry breeding lines. In general, to identify and quantify folates in food, SIDAs combined with LC-MS/MS analysis are advantageous when compared to microbiological assays or LC-UV. In particularly the microbiological assay is not able to determine the vitamer distribution. Ringling and Rychlik (2017a) found distinct differences in total folates between microbiological assays and LC-MS/MS. One of the drawbacks of the microbiological assay was that it may give incorrect results because of different responses to different food matrices.

According to EU-regulations health claims are applicable to strawberries, which contain at least 15% of the nutrient reference value of 200 μg/100 g. In case of strawberries containing at least 30% of the nutrient reference value a health claim “rich in folates” may be used (European Union, 2012). Our results revealed that all strawberries under study except one white breeding line (F. nilgerrensis Mt. Leigong 2) contained more than 30% of the nutrient reference value of 200 μg /100 g and, therefore, can be labeled as “rich in folates.” However, the folate content of strawberries itself does not give any information about bioaccessibility, absorption, retention, and availability for physiological functions. This may depend on many factors such as complete/incomplete release from plant cellular structures, extent of conjugation, vitamer distribution within the food/fruit matrix or the presence of other components such as ascorbic acid. The relative bioavailability of natural folate to that of synthetic folic acid generally is generally estimated to be around 50% (Pentieva et al., 2004). However, a huge variability in folate bioavailability between individuals and even after the consumption of the same food type (Camembert cheese) could be observed in two recently published short-term bioavailability studies with healthy human subjects (Mönch et al., 2015, 2016). Thus, more studies on the absorption characteristics of folates from strawberries in physiological (dietary) dosage are warranted to assess the nutritional value of different strawberry cultivars and breeding lines and to give reliable recommendations for consumption.

In conclusion, the developed and validated SIDA allows the reliable analysis of individual folate vitamers in strawberries and other foods and therefore can help to update and improve dietary recommendations. Although, we could not find a relationship between the folate content and the origin of the strawberries, the present study confirmed previous research that strawberries can be considered as a rich dietary source of natural folates and that this vitamin contributes to the nutritional value of strawberries. However, genotype and environmental conditions can significantly affect the folate content of strawberries and may stimulate the interest in specific breeding programs to increase the natural folate content and subsequently nutritional value of this popular and tasty fruit. Besides, commercial strawberries are often imported goods and a timeframe of a few days from the harvested date until the date of sale has to be calculated. More research should be done on the stability of folates during storage or transportation. Furthermore, folate bioavailability and the development of new strategies to minimize folate losses during storage, transport, and processing should be implemented in future strawberry studies.

## Author contributions

LS, SC, MN, and MR: conceived and designed the experiments. LS and SC: performed the experiments and analyzed the data. MR: contributed reagents, materials, analysis tools. MR, LS, and MN: Wrote paper.

### Conflict of interest statement

The authors declare that the research was conducted in the absence of any commercial or financial relationships that could be construed as a potential conflict of interest. The handling editor declared a past supervisory role with one of the authors, LS.
