# Supplementary material for: Improved Stable Isotope Dilution Assay for Dietary Folates Using LC-MS/MS and Its Application to Strawberries
Source: Front Chem. 2018 Feb 6;6:11. doi: 10.3389/fchem.2018.00011 (PMC5808173; doi:10.3389/fchem.2018.00011)
Supplement: Supplementary file 1 [file DataSheet1.DOCX]

Supplementary Material

Improved stable isotope dilution assay for dietary folates using LC-MS/MS and its application to strawberries

Lisa Striegel*, Soraya Chebib, Michael Netzel, Michael Rychlik

*** Correspondence:** lisa.striegel@tum.de

Development of the method:

The goal to accelerate folate separation with liquid chromatography was intended to be achieved by using a column with core shell particles. However, to ensure that no salt particles and ascorbic acid of the elution buffer enter the mass spectrometer and, therefore, to guarantee sufficient waste time, we first determined the retention time of ascorbic acid and the void time using acetone. With the gradient starting at 5 % B H_4_folate showed no retention and thus we could not ensure enough waste time. By lowering the concentration of B to 3 % followed by raising the concentration to 10 % in 2.5 min we were able to increase the retention time of H_4_folate to 2.3 min compared to the void time of 1.9 min. Then we adjusted the waste time to 2.1 min and assured that interfering substances were eluted within this period.

Optimization of the extraction method:

Freeze dried strawberries were spiked with [^13^C]-5-CH_3_-H_4_folate and a substantial decrease in the recoveries of this vitamer after extraction was found. By adding the extraction buffer first and equilibrating the samples for 15 min no further losses of internal standard were detected.

Calculations of the response equations:

Response equations for the linear functions were calculated as follows (1):

${n(A)}/{n(S)}=m\times{A(A)}/{A(S)}+b$ *(1)*

Response equations for polynomial functions were calculated as follows (2):

${n(A)}/{n(S)}=m_{1}\times{A(A)/A(S)}^{2}+m_{2}\times{A(A)}/{A(S)}+b$ *(2)*
